# A review of PET attenuation correction methods for PET-MR

**DOI:** 10.1186/s40658-023-00569-0

**Published:** 2023-09-11

**Authors:** Georgios Krokos, Jane MacKewn, Joel Dunn, Paul Marsden

**Affiliations:** School of Biomedical Engineering and Imaging Sciences, The PET Centre at St Thomas’ Hospital London, King’s College London, 1st Floor Lambeth Wing, Westminster Bridge Road, London, SE1 7EH UK

**Keywords:** PET-MR, Attenuation correction, Dixon, UTE, MLAA, Atlas-based attenuation correction, Deep-learning-based attenuation correction, Pseudo-CT

## Abstract

Despite being thirteen years since the installation of the first PET-MR system, the scanners constitute a very small proportion of the total hybrid PET systems installed. This is in stark contrast to the rapid expansion of the PET-CT scanner, which quickly established its importance in patient diagnosis within a similar timeframe. One of the main hurdles is the development of an accurate, reproducible and easy-to-use method for attenuation correction. Quantitative discrepancies in PET images between the manufacturer-provided MR methods and the more established CT- or transmission-based attenuation correction methods have led the scientific community in a continuous effort to develop a robust and accurate alternative. These can be divided into four broad categories: (i) MR-based, (ii) emission-based, (iii) atlas-based and the (iv) machine learning-based attenuation correction, which is rapidly gaining momentum. The first is based on segmenting the MR images in various tissues and allocating a predefined attenuation coefficient for each tissue. Emission-based attenuation correction methods aim in utilising the PET emission data by simultaneously reconstructing the radioactivity distribution and the attenuation image. Atlas-based attenuation correction methods aim to predict a CT or transmission image given an MR image of a new patient, by using databases containing CT or transmission images from the general population. Finally, in machine learning methods, a model that could predict the required image given the acquired MR or non-attenuation-corrected PET image is developed by exploiting the underlying features of the images. Deep learning methods are the dominant approach in this category. Compared to the more traditional machine learning, which uses structured data for building a model, deep learning makes direct use of the acquired images to identify underlying features. This up-to-date review goes through the literature of attenuation correction approaches in PET-MR after categorising them. The various approaches in each category are described and discussed. After exploring each category separately, a general overview is given of the current status and potential future approaches along with a comparison of the four outlined categories.

## Introduction

The combination of two of the most established methods in patient care such as positron emission tomography (PET) and magnetic resonance imaging (MRI) could potentially provide invaluable complementary functional and anatomical information. Lower radiation delivered to the patient, improvements in image quality mainly due to advancement in motion correction techniques and benefits in radiotherapy planning due to more accurate target delineation are just some of the benefits already provided [1, 2]. The first commercially available PET-MRI systems though were introduced more than a decade ago, and despite the initial excitement in terms of how the systems could revolutionise molecular imaging, they are still not widely used in routine clinical practice. One of the main reasons is the reported discrepancies in tracer uptake, prompted by the vendor-provided attenuation correction (AC) methods, when compared with more established techniques such as computed tomography (CT) or a transmission scan, which may hamper accurate quantification. CT and transmission scans are based on the attenuation of photons in the medium, which can be directly exploited for correcting the PET images. If the CT-based AC values are appropriately converted to 511-keV linear attenuation coefficients, the method provides highly accurate results for reconstructing PET data [3]. The signal intensity in MRI, however, is not representative of tissue density or the atomic number of the imaged material, which makes the definition of an AC map more complicated. Tissues that do not provide an MRI signal such as bone and air will lead to errors in bony structures or lesions near bone in the reconstructed PET images. Moreover, involuntary motion has always been, and remains, a challenging issue in the concept of attenuation correction, and PET in general, while subject specific differences in densities for certain organs such as the lung, may constitute the use of global attenuation correction factors as an ill-advised technique.

**Fig. 1:**
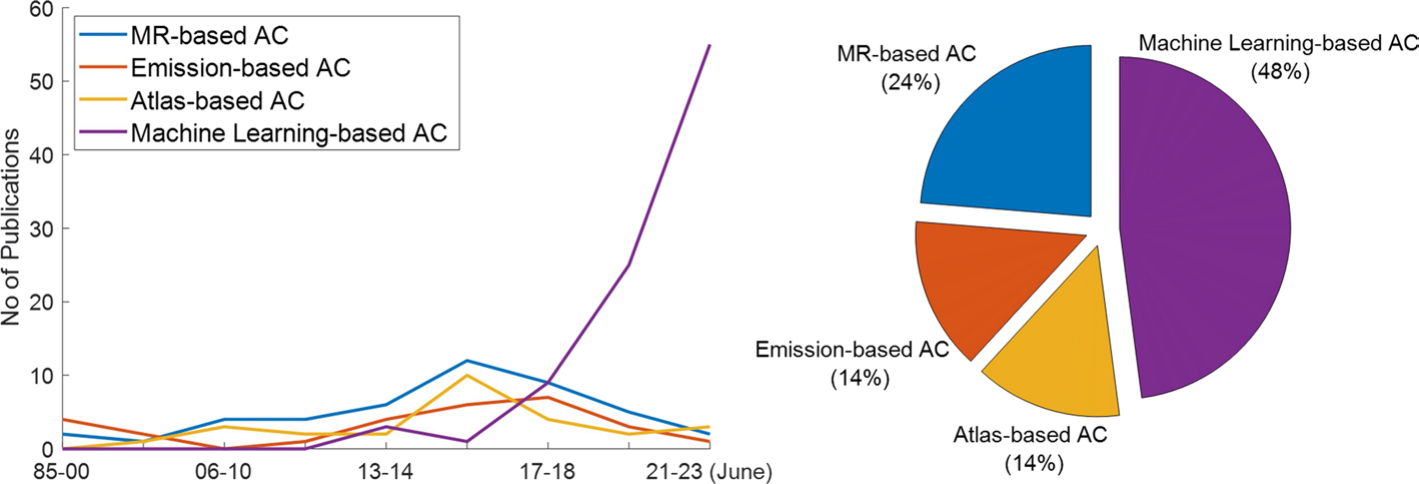
Number of publications from 1985 to 2023 (June) introducing a new technique for attenuation correction for PET-MR data. The pie chart indicates the proportion each group of methods (MR-based, emission-based, Atlas-based and machine learning-based AC) occupies in all literature included in the left plot. For the generation of this figure, the keywords “PET-MR” and “attenuation correction” were used in Google Scholar and PubMed. All results were then filtered to identify studies introducing a new method

As a result, ongoing attempts from the scientific community to address the problem as accurately as possible have led to an extensive number of publications describing a very wide range of proposed AC techniques [4–11]. In Fig. 1, the increasing number of proposed techniques over the years can be appreciated along with how machine learning methods have within a few years outnumbered all other methods.

The existing literature can be broadly partitioned into the following categories:

1. MR-based AC (MRAC): The direct use of MR sequences which aim to extract information regarding the attenuation properties of the tissues.
2. Emission-based AC: The direct use of emission PET data to predict the AC map.
3. Atlas-based AC: The generation of a pseudo-CT using databases of PET, CT, MRI and transmission images.
4. Machine learning-based AC: A collection of machine and deep learning techniques which exploit databases of mainly MR, CT and PET data to identify underlying correlated features.

This review will describe each one of the above categories, along with all recent advancements, while the benefits and disadvantages of those methods will be discussed. Rather than focusing on a specific organ or method, an overall view of all proposed techniques will be given. A handful of methods not falling under one of the pre-described categories will be separately discussed. At the end, a general discussion of the current status and the potential future direction of attenuation correction in PET-MR will be presented.

## Motion artefacts

Motion is inextricably intertwined with attenuation correction. However, since motion correction is a large and active field of research, we will not include the details of the various motion correction methods. Instead, we advise the reader to refer to comprehensive reviews covered in [12–14]. We do, however, need to briefly comment on some of the specific issues that motion causes on PET-MR acquisitions. In general, motion during MR data acquisition results in corrupted k-space data leading to artefacts such as ghosting, blurring and others [14], which can subsequently have a direct effect on the attenuation correction of the associated PET images. In most vendor-provided MRAC techniques, in order to minimise motion due to respiration which is the main contributor to motion artefacts, the patient needs to hold their breath during the acquisition [15], which, despite the difficulties it poses for certain clinical conditions, can still result in misaligned PET and MR or CT images and, conversely, in artefacts on the final PET images [16]. In practice, it is also quite common that the patients might hold their breath at end-inspiration rather than end-expiration or vice versa leading to considerable biases on the PET images [17]. Moreover, involuntary motion of abdominal organs, although more subtle, is difficult to address and can also lead to misregistration errors [14]. In clinical PET-CT examinations, some of the challenges in cardiac and lung imaging can be overcome by allowing free breathing and averaging the dynamic CT images [18] or even a static CT image during free-breathing seems to be quite insensitive to misalignment errors [19]. As mentioned, such approaches in MR imaging could create a phase difference (and therefore, ghosting artefacts) while populating the k-space rather than just simply producing an averaged image. Various other methods have been proposed in order to make MR acquisitions less prone to motion artefacts such as radial sampling of the k-space [20], gating of the MR signal [21], the use of MRI-derived motion fields to perform motion correction [22, 23] in combination with anatomically guided PET image reconstruction [22], accelerated techniques to avoid breath-hold [24, 25] and more. Specifically for this review, most studies attempt to validate the proposed method on the PET-MR using a separately acquired CT, which is brought in the MR space. In the atlas and machine learning methods, pairs of CT and MR data are employed for predicting the final image used for attenuation correction (more details in the corresponding sections). Involuntary motion in non-rigid organs though such as the lungs, heart and bowels, renders coregistration between the two images challenging. Although most studies tend to apply rigid followed by non-rigid registration, small levels of misalignment may still be observed at the edges of organs, which might be mistaken as a “disagreement” between the two methods in the attenuation-corrected PET image [26, 27].

## MR-based attenuation correction

### Vendor-provided techniques

The majority of vendor-provided techniques for AC are based on the 2-point Dixon method [28], which uses two different echo times, taking advantage of the slightly different precession rates of fat and water molecules to create an image. This image can then be classified into soft tissue and fat, and along with the background and lung, pre-defined attenuation coefficients (*μ*) are assigned. The first obvious problem with this method is that bone and lung tissue do not produce an MR signal and therefore cannot be distinguished in the images due to both having extremely short *T*_2_*. This causes a bias in corrected PET images in terms of standardised uptake values (SUVs), which has been quoted to range between 10 and 30% in soft tissue and even more in bone lesions, compared to “gold standard” methods such as CT attenuation correction (CTAC) or transmission scans [29–45], even though it can still be useful for bone lesion identification if quantification is not of interest [46]. In a whole-body study, Izquierdo-Garcia et al. [47] reported differences of more than 10% in the spine, lung and heart with the MRAC method being also susceptible to metallic artefacts and artefacts due to the limited MR field of view (FOV), which truncates parts of the body located outside of it, also known as truncation artefacts. To tackle truncation artefacts in the body, the “*B*_0_ homogenisation using gradient enhancement" (HUGE) method was proposed and implemented on the Siemens mMR scanners, a sequence technique which results in an extended FOV [48, 49].

In order to incorporate information about bone tissue in the *μ*-maps, a method that superimposes bone tissue on the Dixon-generated *μ*-maps was introduced, using intensity- and landmark-based deformable registration between an atlas consisting of MR images and bone mask pairs and the patients’ Dixon image (SEGBONE method) [30]. This significantly decreased bias in brain even though a considerable number of outliers were still present [36]. Even though the SEGBONE method shifted SUV values in the body in the correct direction [50], significant bias is still reported in lung and spine [30]. However, minimal effects were reported in prostate [51].

An alternative but popular approach is the use of the ultra-short time echo (UTE) sequence, which is acquired at approximately 100 times shorter echo times compared to most anatomical *T*_1_-weighted MR images (will be referred to as *T*_1_*w* for the rest of this review) and could capture the signal from regions with very short *T*_2_* such as bone [52]. In short, this is achieved by using data from two very short (or half) pulse excitations with inversed polarity and spiral mapping of the k-space. A number of methods to make acquisition faster by either under-sampling *k*-space, switching the readout gradient earlier, and modifying the dual to a single echo acquisition have also been proposed, which provide results comparable to the original UTE [53–58]. Despite its popularity for attenuation correction in PET, a number of studies have reported significant underestimation in PET SUV values in the brain, ranging between 4 and 17% when compared to CTAC, especially in the cortical regions [29, 36, 59–61], and misclassification of voxels belongs to the ventricles, which were classified as air [62], and bone, which was classified as tissue [59, 61, 63, 64]. In the lung, UTE performs well in terms of tissue detectability [65, 66], but the sequence has not been extensively applied in the body due its long acquisition time [67]. It has also been demonstrated that the change in the magnetic field during the UTE sequence induces eddy currents that lead to degradation of the reconstructed images and misclassification to tissue boundaries [68].

**Fig. 2:**
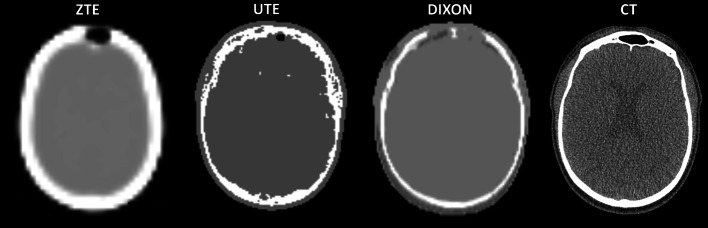
Various vendor-provided MR attenuation correction methods along with a low-dose CT used for CT attenuation correction

The zero time echo (ZTE) sequence provided on the GE SIGNA is based on the same principle as UTE with the difference that the readout gradients are turned on before the radiofrequency excitation and encoding starts at the same time as signal excitation making it possible to acquire an image with almost zero TE [69]. The bone regions from this method were found to have a high degree of overlap when compared to the regions from the corresponding CT images although misclassification of dense bone tissue as air was also reported [70]. When directly applied for attenuation correction of PET data though, the results in the literature range from marginal SUV differences when compared to CTAC [71, 72] to overestimations of up to 10% [73–77], especially in the cerebellum. In the lung, ZTE has shown promising results in terms of contrast and lesion detectability [78, 79]. However, no studies performing a quantitative evaluation of the method in the body were found. More recently, Engström et al. [80] provided some insight on the fat–water chemical shift artefact, which is often apparent in ZTE images (a non-uniformity artefact mainly prominent in tissue edges) and leads to tissue misclassification. All manufacturer-provided methods for a single patient are presented in Fig. 2 along with a CT image for comparison.

### Adaptations of vendor-provided techniques

#### Tissue segmentation

Even though the MRAC methods outlined above were reported to have discrepancies with the more established CTAC and transmission scans, their ability to identify certain tissue classes and their ease of use motivated a number of studies to further improve them.

An obvious approach would be to combine either the readily available [81, 82] or modified versions of the UTE and Dixon sequences [57, 83–85] to improve classification accuracy to the various tissue classes. For example, Su et al. [83] used the UTE to discriminate air and bone along with the modified DIXON sequence, which includes a flexible choice of echo time pairs rather than fixed values, for better differentiation of fat and water tissue while Han et al. [85] combined UTE with 6 multi-echo DIXON sequences to enhance tissue differentiation.

Although the implementation details differ between the various studies, an improved tissue class identification is reported when compared to either the Dixon, UTE or the ZTE alone in the brain [81, 85] the thorax [83] or the pelvis [82]. Alternatively, a few studies suggest an improvement of tissue classification by extracting information regarding the tissue properties from conventional anatomical sequences such as *T*_1_ images [39, 86], *T*_1_ and *T*_2_ maps [87], combinations of multiple turbo field-echo sequences [88] or a ^31^P-MRI image to utilise the signal from the phosphorus atoms present in the bone crystals [89]. A rigorous assessment on clinical PET data still needs to be performed for those methods, while the main limitation is the long acquisition time needed for all required sequences to be acquired.

The use of intermediate UTE images for a more accurate segmentation of air, bone and soft tissue has been proposed by a number of studies [61, 90, 91], which reported a significant decrease in SUV bias compared to the original UTE and an approximate 5% bias in the majority of the brain when compared to CTAC. Most notably, Ladefoged et al. [92] extracted the air, bone and whole brain volume using UTE images, while CSF and brain tissue were identified by registering the images to the structural template provided by the Montreal Neurological Institute, space (MNI) in what is known as the RESOLUTE method.

To explore possible limitations in using uniform μ values in bone tissue, Khalife et al. [73] suggested segmenting the bone region and applying continuous CT-derived values by using a linear relationship between normalised ZTE and CT signal intensity. However, the increase in accuracy was relatively marginal when compared to using uniform values in the bone. A number of other methods have also focused on the accurate classification of the bone region [93–95] and the correct assignment of the μ values within the classified bone region [93].

#### Metallic artefacts

Besides tissue segmentation accuracy, the other great challenge in MRAC techniques is to address artefacts caused by metallic implants and properly accounting for any hardware in the FOV. In an attempt to mitigate susceptibility artefacts caused by metallic implants, Burger et al. [96], combined the Dixon images with the multi-acquisition variable-resonance image combination and slice encoding for metal artefact correction. Although their study showed promising results, the sequences are still quite long to be easily incorporated in a clinical PET-MR examination. Alternatively, Ladefoged et al. [97, 98] and Schramm et al. [99] used the PET along with the MR images to identify and segment the implants before incorporating them into the *μ*-map, which resulted in the mitigation of gross artefacts in the final images.

Currently, the *μ*-map of most of the scanners’ hardware is already incorporated in the latest MRAC methods [100] even though additional components such as the headphones, radiotherapy beds or body-coils could still lead to considerable bias [101] and any additional hardware needs to be accounted for [102]. Additionally, it has been shown that discrepancies of up to 3 cm between the actual position of the coils over the patient’s scanned area with the scanner-defined one could also lead to 10% bias in the mean SUV value [103, 104]. Manually adding any additional hardware on the default vendor-provided *μ*-map after scanning it on CT [104] or by using computer-aided design models rather than a CT scan [105] could lead to substantially improved accuracy in the PET images.

### Alternative MR-based attenuation correction methods

As mentioned earlier, one of the main challenges with attenuation correction in PET-MRI is that there is no direct relationship between CT and MR signal [6]. There have been several attempts trying to correlate the information from the two modalities in an effort to confidently create a *μ*-map by exploiting the various imaging techniques provided by the MRI. Delso et al. [106] reported that a one-to-one relation between CT and the transverse relaxation rate (*R*_2_^*^) MR images was difficult to establish but the latter did contain a certain level of anatomical information of the bone, which could potentially be utilised. Moreover, good correlation was reported between CT HUs and an anatomical *T*_1_ [107] or a combination of *T*_1_ and *T*_2_ images [108] for bone and tissue even though organs susceptible to involuntary movement such as the bowel and bladder and bony-tissue interfaces were still not accurately defined [108].

Alternatively, rather than trying to establish a correlation in the signal intensities with CT, a few studies tried to directly employ MR sequences (other than the vendor-provided MRAC methods) for tissue segmentation. A popular approach is the use of fuzzy C-means clustering either on *T*_1_ [109, 110], on UTE [57, 111], on time resolved-angiography [112] or a combination of anatomical and UTE images [113], which led to promising results with good agreement between the reconstructed images of the proposed methodology and the reference method. However, the systematic overestimation of SUVs in soft tissue and underestimation in bone was still an issue [109, 112].

All studies that introduced a new method and were evaluated on clinical PET data against a reference method for attenuation corrections are listed in Table 1. For better clarity, only studies with a reported relative error are outlined and a selection of anatomical regions on which they were evaluated, if multiple, are mentioned.

**Table 1:** List of original MR-based methods evaluated on clinical PET data

Study	Method	Validation data† (Tracer)	Region	Reported error (%)	
				Proposed method	Vendor method
Zaidi et al. [109]*	Segmentation of *T*_1_ images by means of fuzzy clustering into air, skull, brain tissue and nasal sinuses	10 (2-[^18^F]FDG)	Brain	~ 4.2 (cerebellum)	N/A
Keereman et al. [90]	*R*_2_ maps derived from UTE sequence to perform three tissue classification (bone, soft tissue and air)	5 (2-[^18^F]FDG)	Brain	5 ^□^	N/A
Cabello et al. [61]	*R*_2_ maps derived from Dual-echo ultra-short time (dUTE) sequence to estimate *μ*-map	9 (2-[^18^F]FDG)	Brain	−0.87 ± 2.1	Dixon: −14.8 UTE: −13.5
Juttukonda et al. [81]	Use of dUTE and 2-point Dixon to segment air, bone with continuous μ values, fat and soft tissue (CAR-RiDR)	98 ([^18^F]Florbetapir)	Brain	2.55 ± 0.86 ^^□^	N/A
An et al. [116]	Two-phase level-set segmentation applied on the intermediate dUTE images to identify air, bone and soft tissue	10 (2-[^18^F]FDG), 20 ([^18^F]FP-CIT)	Brain	9 for 2-[^18^F]FDG, 4 for ^18^F[FP-CIT] (cerebellum)	UTE: −35 UTE: 14
Ladefoged et al. [92]	UTE sequence to segment air, bone and brain volume and MNI template to segment CSF regions (RESOLUTE)	164 (2-[^18^F]FDG)	Brain	−1.2	Dixon: ~ −12.5 UTE: ~ −7.5
Hu et al. [117]	Segmentation of a 3D multi-station spoiled gradient echo sequence into air, lungs, soft tissue, and bone regions using intensity and a-priori knowledge of the organ anatomy	15 (N.A)	Whole body	−7.6 ± 7.5 (bone lesions) −4.4 ± 3.8 (lung lesions) −6.4 ± 10.2 (heart)^	N/A
Schulz et al. [86]	Segmentation of *T*_1_ images to air, lung and soft tissue	15 (2-[^18^F]FDG)	Whole body	6.9 ± 9.5 (Soft tissue lesions),3.0 ± 3.9 (bone lesions), 1.9 ± 5.8 (lung lesions) ^▪^	N/A
Marshall et al. [37]	Segmentation of a Turbo-FLASH sequence to air, lung, fat and soft tissue and addition of bone from information from a CT atlas	12 (2-[^18^F]FDG)	Whole body	1.3 ± 0.9 (bone) 8.0 ± 2.9 (lung) 2.6 ± 0.4 (soft tissue) ^	N/A
Paulus et al. [30]	Addition of bone in Dixon images using a bone atlas (SEGBONE)	20 (N.A)	Whole body	−4.9 ± 6.7 (bone) 2.9 ± 5.8 (bone lesions)^	Dixon: −46.5 ± 6.7 Dixon: 7.3 ± 5.1^
Leynes et al. [82]	Combination of 2-point Dixon and ZTE sequence to segment air, bone with continuous *μ* values, fat and soft tissue	6 (2-[^18^F]FDG)	Whole body	−3.2 ± 0.8 (bone) −3.5 ± 1.7 (bone lesions)^	Dixon: −10.8 ± 2.2 Dixon: −7.7 ± 0.8^

The regional mean relative error along with the standard deviation (where available) in radiotracer uptake across subjects is reported unless otherwise specified. The corresponding error for the vendor provided method is quoted where available. CT was used for reconstruction of the reference images unless otherwise specified

^†^Number of patients on which the method is evaluated, *transmission data used for reconstructing the reference PET images, ^ relative absolute error is reported, ^▪^relative difference in SUV_max_ is reported, ^□^ voxel-wise error is reported

### Discussion

#### Brain

Despite the popularity of the vendor-provided MRAC techniques, various evaluation studies have demonstrated that they might lead to high biases when compared to a CTAC, mainly due to the lack of bone information in the Dixon sequence and voxel misclassification in both. The majority of the available studies focus on brain as most of the proposed sequences are too long for whole-body applications [86, 114]. Moreover, the head is not hampered by additional sources of error such as truncation artefacts, while patient motion can be more easily regulated [115]. Consequently, a rich literature of various methods, which can outperform the ones supplied by the manufacturers, is already available [30, 37, 61, 81, 82, 86, 90, 92, 109, 116, 117]. The addition of bone in the Dixon and the ZTE sequence seems to provide much more promising results in the brain, although careful assessment of the cerebellum and cortical regions is still needed [36, 90]. Current studies have also indicated that no substantial difference is noticed in the PET-reconstructed images when using fixed or continuous μ values for bone tissue [73]. A more synergistic technique between the vendor-provided methods could be the most straightforward approach to increase accuracy, but an evaluation in whole-body PET is still required [81, 82].

#### Whole-body

The areas that seem to provide the least accuracy in the reconstructed PET images are the lung, bone, bone lesions and the heart, even after the introduction of SEGBONE in the Dixon *μ*-map. In the lung, where high discrepancies are reported, the problem seems to be more convoluted. Various studies have reported that the density is quite variable and volume, age, sex and smoking status dependant, while density difference due to the respiratory stage could induce errors as high as 30% [118, 119]. The reported true linear attenuation coefficient values range between 0.018 and 0.027 cm^−1^, which can have a considerable impact in the PET SUV [6]. Beyer et al. [120] also quoted differences of up to 20% just by comparing the linear attenuation coefficients between vendors, indicating that some level of standardisation is required. In addition, when applied to simulated PET data, underestimations of up to 50% were reported, significant errors when truncation artefacts are present while imperfect registration between PET and MRAC or CTAC (see motion correction section) could lead to 20% bias in SUV [26]. Moreover, it has been reported that iron overload in certain patients could also lead to misidentification of liver tissue as lung [121].

Ideally, a simple and fast MRI-only method that is applicable in whole-body scans for accurate attenuation correction would be provided. Alternative methods using multiple MR sequences might be of interest but still need to be validated. However, the long acquisition times render them impractical for clinical PET-MRI applications.

## Emission-based attenuation correction

### The maximum-likelihood reconstruction of attenuation and activity algorithm (MLAA)

A rather appealing approach is to try and generate the *μ*-map during the reconstruction process based on the PET emission data without additional acquisition of a *μ*-map. Some of the earliest approaches included the use of the emission data for finding the various head regions on which a uniform *μ* value [122] was applied, and then combine with the information from the emission and transmission scans using a joint objective function during the reconstruction [123, 124], or the application of discrete consistency conditions on the data [125–128]. The most popular method currently is the maximum-likelihood reconstruction of attenuation and activity algorithm (MLAA) [129]. The basic concept is to incorporate the reconstruction of the *μ*-map in the process of iterative reconstruction of the PET data. The radioactivity concentration is estimated in each iteration for the reconstruction of the PET image while keeping the *μ* values constant as it would be normally done in iterative image reconstruction. Each iteration for the PET image is followed by an update (iteration) of the *μ*-map during which the radioactivity concentration remains constant in this intertwined iterative procedure. As the emission data need to provide a level of information of the attenuating medium, this method is mainly used in conjunction with time-of-flight (TOF) as non-TOF systems result in crosstalk artefacts (between activity and *μ*-maps leading to reduced *μ* values in regions of high activity) and high noise [130, 131]. Initial studies provided encouraging results in terms of image quality, while the method was able to compensate to some extent for truncation artefacts [129, 130, 132]. However, it has been shown that the *μ* value can only be estimated up to an additive constant, which can be problematic when quantification is of interest [132]. Moreover, the low count bias present in the MLEM/OSEM algorithm seems to be further exacerbated when the MLAA algorithm was applied [133] rendering the method inappropriate for dynamic studies with low count frames. The combination of multiple attenuation maps from dynamic data generated with the MLAA algorithm was shown to moderately improve the estimation of a single map in terms of accuracy but did not address the limitations described above [134].

#### Tackling the additive constant in MLAA

A number of methods have been proposed in order to address the limitations of the additive constant and noise in the early MLAA approaches. Salomon et al. [135] suggested the use of MRI images with organ segmentation to update the μ values in the image in a regional rather than a pixel-wise basis. Boellaard et al. [136] demonstrated that this method reduces bias in bone regions from approximately 50–15% when compared to Dixon MRAC methods and better addressed the truncation artefacts. The average bias in lesion SUV values in clinical data was also reduced, but a high variance was observed. Moreover, since *T*_1_ anatomical images cannot distinguish bone from air, many voxels in air cavities were misclassified and the *μ* values for bone were underestimated [137]. To further increase the accuracy of segmentation and reconstructed PET images, a few similar methods have been proposed using a tissue prior atlas [138], an MR-based AC image instead [139–142], combination of *T*_1_ and UTE images [143] or anatomical *T*_1_ images along with penalisation functions in the MLAA for estimating the PET attenuation-corrected image and *μ*-map [133] and more [142, 144]. Most of those methods report an error of < 7% in the brain which is more than a twofold lower compared to UTE, two-point Dixon and Salomon’s method.

Another attractive advantage of the MLAA technique was the potentially accurate reconstruction of the lungs since misregistration artefacts due to breathing motion could be avoided as there would not be a need for an anatomical image. Most of the aforementioned techniques that attempt to address the additive constant introduce an anatomical image, while most methods performed poorly in air cavities due to voxel misclassification. Attempts to reconstruct the lung while tackling the additive constant issue include lung segmentation within the MLAA reconstruction process [141], the use of non-attenuation-corrected images (NAC) [145] or CT images [137] to segment the lung prior to the final reconstruction. However, high biases in lung edges probably caused by imperfect segmentation [145] and the need for CT scans [137] indicate the requirement for these methods to be further developed to be of practical use in a clinical PET-MR facility.

### Alternative emission-based methods

The two main alternatives to the MLAA method are the maximum likelihood activity reconstruction and attenuation correction registration (MLRR) [130, 131, 146], and the maximum-likelihood activity and attenuation correction factors estimation (MLACF) [147]. In the MLRR, proposed by Rezaei et al. [130, 131, 146], a CT image from a previous scan of the patient is included in the reconstruction process and instead of updating the μ values, those are considered known and the deformation field between PET and CT is updated. Although this method seems to provide promising results, it is more meaningful in the non-rigid regions of the body and it requires the existence of a CT scan of the patient. Moreover, the change in density between respiration phases in the lung is not taken into account [6]. The MLACF method on the other hand simplifies the MLAA method by only updating the radioactivity concentration during iterative reconstruction, while the μ values are calculated by a closed-form solution [147, 148]. The simpler reconstruction process makes this method faster than the MLAA but since no anatomical reference is incorporated, and an overall non-negativity constraint of the attenuation correction factors is applied instead, the images are noisier especially in low count regimes [148]. Moreover, prior information regarding the tracer distribution, such as known amount of activity in the FOV, needs to be provided, which might be impractical in clinical practice. However, promising results were provided when applied on brain data with errors lower than 4% [149] and good performance even in systems with limited FOV [150].

Finally, although not strictly falling under this category, it is worth mentioning that a small number of studies attempted to generate the attenuation map using scatter [151–153]. Those studies have drawn limited attention so far probably because they have mainly been evaluated on simulated data [154].

All emission-based AC methods that have been applied on clinical PET data and report relative agreement with a reference method are listed in Table 2.

**Table 2:** List of original emission methods evaluated on clinical PET data

Study	Method	Validation data† (Tracer)	Region	Reported error (%)	
				Proposed method	Vendor method
Siegel et al. [122]*	Segmentation of body contour from sinogram data	~ 300 (2-[^18^F]FDG, [^18^F]DOPA, [^15^O]H_2_O and [^13^N]Ammonia)	Whole body	< 8.9% (Brain 2-[^18^F]FDG), ± 10% (renal flow ([^13^N]Ammonia)	N/A
Chang et al. [145]	Segmentation of body organs from NAC PET images	14 (2-[^18^F]FDG)	Thorax	6 ± 7 (lung lesions), 3 ± 6 (bone lesions) ^▪^	N/A
Mehranian et al. [142, 304]	MLAA with prior MR spatial and CT statistical information using Gaussian mixture model (MLAA-GMM)	5 (2-[^18^F]FDG)	Whole body	−11.6 ± 6.0 (cerebrum), −3.5 ± 6.6 (lung), −12.6 ± 8.6 (bone)	Dixon: −18.5 ± 11.3, Dixon: −5.4 ± 12.0, Dixon: −19.8 ± 8.4
Mehranian et al. [137]	MLAA-GMM including lung segmentation from CT images	17 (2-[^18^F]FDG and 2 with [^18^F]Choline)	Whole body	−0.8 ± 6.3 (lung), −5.7 ± 3.2 (myocardium), −2.0 ± 2.1 (bone)	Dixon: −5.2 ± 7.1, Dixon: −9.5 ± 4.3, Dixon: −14.1 ± 2.5
Benoit et al. [143]	MLAA using UTE and T_1_ images as anatomical prior information for updating the attenuation map	204 (2-[^18^F]FDG)	Brain	−1.5 ± 3.5	Dixon: −11.8 ± 4.5, UTE: −6.5 ± 2.0
Mehranian et al. [133]	MLAA with spatial constrains from MR images using penalty functions during image reconstruction for updating μ values (P-MLAA^+^) and both *μ*-map and activity distribution (P-MLAA^++^)	8 (2-[^18^F]FDG)	Brain	−3.0 ± 3.5 (P-MLAA^+^), −4.2 ± 3.6 (P-MLAA^++^)^□^	Dixon: −13.5 ± 3.1

The regional mean relative error along with the standard deviation (where available) in radiotracer uptake across subjects is reported unless otherwise specified. The corresponding error for the vendor provided method is quoted where available. CT was used for reconstruction of the reference images unless otherwise specified

^†^Number of patients on which the method is evaluated

*transmission data used for reconstructing the reference PET images, ^▪^relative difference in SUV_max_ is reported, ^□^ voxel-wise error is reported

### Discussion

The emission-based methods seem very efficient, as in principle no information regarding tissue density is required. Moreover, these methods address the misregistration problems between PET data and attenuation maps which are of particular issue in the lungs and heart. By far, the most popular method is the MLAA. However, in order to avoid crosstalk artefacts and excessive noise in the images, it could only be implemented on systems with TOF capability. Variations that claim that this method could be confidently applied in non-TOF systems have mainly been evaluated in the brain where TOF does not have as big an impact as in the rest of the body especially in the thorax where the crosstalk artefact could lead to excessive biases [155, 156].

Another issue that needs to be tackled with this method is that of the additive constant. Most techniques employed to address the problem use anatomical priors from MR images. Nevertheless, a few studies indicate that it is still not fully addressed in whole-body regions [131, 142]. A more in-depth look at the inherent problems of the MLAA reconstruction algorithm, including the additive constant issue and problems related to convergence and dealing with voxels of zero value, is given by Salvo and Defrise [157]. MLAA seems to be able to overcome the truncation artefacts present at the edge of the FOV and is currently provided in the Siemens mMR scanner in combination with the Dixon-MRAC to fill the missing information. However, the more recent MR-only HUGE method seems to be outperforming MLAA for that purpose [158].

Most emission-based methods are also dependent on the timing resolution of the scanners [130, 132, 159, 160]. Therefore, even though currently they might still be considered as methods in development, it may be the case that in the near future, with continuous advancements in the PET system electronics [159], their performance will improve.

## Atlas-based attenuation correction methods

The main concept of the atlas-based methods is to predict the required image for attenuation correction (e.g. CT) from the available image acquired from the PET-MR scan (e.g. an anatomical MR). This is done by generating a database of one or more of the required images from the general population and employing registration techniques between the available image from a new subject and the images in the database. The concept of constructing an atlas of anatomical images is not novel but has been around for more than 35 years [161, 162]. Therefore, one of the potential advantages of this method is that it is not a revived method such as the Dixon sequence or the emission-based reconstruction but has been used routinely in different contexts to attenuation correction, and as a result, it has been evolved and optimised over the years. In its earlier applications, this method would only use a single or an averaged image (reference) rather than a whole dataset. The accuracy of the method would then be highly dependent on the accuracy of the registration of the reference image to the corresponding image of the new patient (target). These were evolved to the more widely used multi-atlas method in which multiple images from a population are available for application on the target image which improves registration accuracy by accounting for inter-subject variability [163]. A popular sub-category of the latter is the registration of database images to the same stereotaxic space (template), to generate a probabilistic map. The target image is then registered to the template and the probability that an area or voxel belongs to that particular class is estimated. Finally, to further improve registration between different modalities, the dual and triple multi-atlas methods were introduced with a database of pre-aligned images, e.g. CT and MR pairs with each pair acquired from the same subject. The MR images in the previous example would act as “intermediary” images to perform registration between the reference in the database and the target to eventually identify the corresponding CT image [5].

In the context of this review, the task is to estimate an accurate image that can be used for attenuation correction (such as a CT image) by registering the atlas to an anatomical MR image of the subject before applying it on the PET data. The main differences between methods are the type of images constituting the atlas (transmission data, CT images, MR images, etc.) or the type of atlas (single-atlas, multi-atlas, dual, etc.). Studies using pairs of transmission data with AC PET images have had limited attention as different radiotracers result in different biodistribution and therefore AC PET images, leading to the requirement for tracer-specific databases. Moreover, the methods did not perform better than when anatomical images were used instead [164–168].

Even though many studies trying to add bone information in the images using an atlas can be considered part of this category, those have been already mentioned in the other sections and we will only describe studies where the whole attenuation map is constructed using an atlas method.

### Anatomy-based atlases

**Fig. 3:**
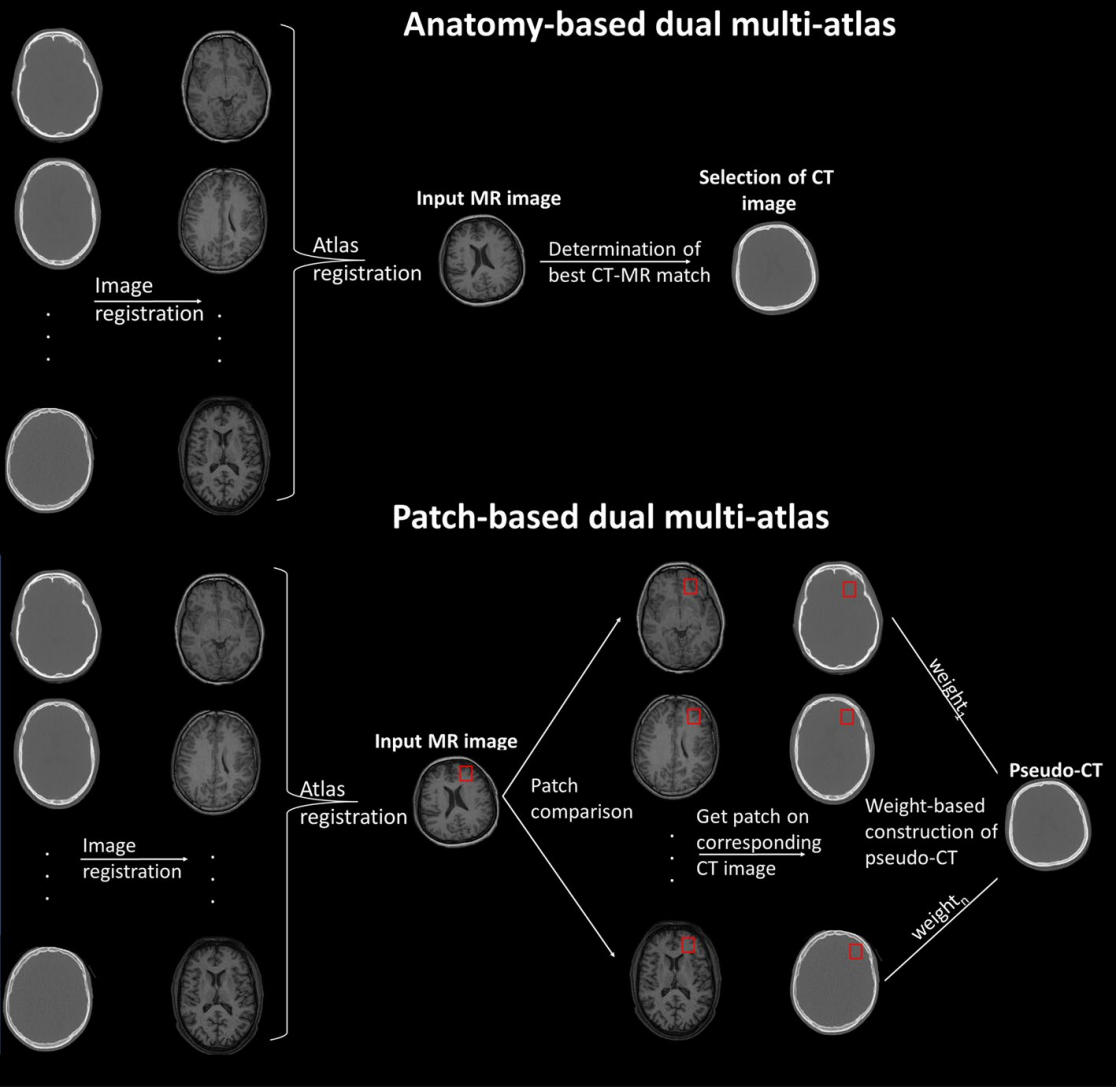
Examples of the general principle of the anatomy- and patch-based dual multi-atlases

In the most straightforward approach, an averaged CT can be created by selecting a representative subject and registering the rest of the CT images in the database before averaging all images. The averaged image is registered to the target to create the pseudo-CT in this single-atlas method [169]. Such an example is readily available on the GE SIGNA scanners, with the CT atlas applied on the patients’ *T*_1_ image and has exhibited bias of less than 8% in reconstructed 2-[^18^F]FDG brain images [71, 170]. Since in PET-MR scans an MR anatomical image is usually available, most studies employ dual multi-atlas techniques with coregistered CT and MR images [171] an example of which can be seen in Fig. 3. Alternatively, statistical parametric mapping (SPM, https://www.fil.ion.ucl.ac.uk/spm) can be used to create a CT- and MR-template of tissue classes with the latter now being the “intermediary”. The target’s intensity-normalised *T*_1_ image is segmented into a tissue map and registered to the MR-template before the inverse transformation matrix is applied to the corresponding CT-template [172]. The use of dual-echo UTE as target images to coregister with the *T*_1_ atlas [173] or the direct use of the *T*_1_ template to classify tissues and assign uniform *μ* values [174] have been proposed. More recently, in order to also make the method applicable to PET only scanners, Jehl et al. suggested the use of PET- and CT-templates with the PET template being registered to the target’s non-attenuation-corrected PET data and the transformation matrix applied on the CT template [175].

### Patch-based atlases

#### Brain

Rather than finding the best candidate from the CT-atlas, the most commonly used methods attempt to create a completely new pseudo-CT image by selecting sections of the brain called patches (which can be as small as a voxel) and trying to find the best candidate for that particular section. This is repeated for all sections of the image and the methods differ on the identification of the optimum patch and on the calculation of the HUs from the atlas database.

In such an example, Burgos et al. made use of a dual-atlas with voxel-level patches being used to assess the similarity of the target’s MRI with the MRIs from the MR-atlas. Weight-based averaging was then applied to the images of the CT-atlas to estimate the HUs on the final pseudo-CT [60, 176]. The proposed method performed better than the UTE, especially in the cortex, and provided good correlation with PET images reconstructed using patient-specific CT [177]. Another notable study by Merida et al. [178] employed majority voting to determine the tissue class and the pseudo-CT voxels were generated by averaging the HUs of the voxels belonging to the same class from all CTs in the database in a method known as MaxProb. The method exhibited biases of less than 5% in tissue of reconstructed brain PET images with various tracers [179, 180].

A few more sophisticated methods have also been proposed which include sparse regression to match the target patch with the MR-template patches after segmenting the air [181], a Bayesian framework to combine patches between CT-UTE atlas pairs [182] and more [183–185]. Nonetheless, even though the added complexity for many of those methods resulted in reconstructed images with relatively low bias, they were still not more accurate when compared to more straightforward methods, which were previously described [176, 178, 185]

#### Whole-body

Atlas-based methods are limited in whole body. For the thorax, Arabi and Zaidi proposed to use a reference patient and precomputed the transformation matrices after coregistering the rest of the MR-CT pairs on that patient. The target MR image would need to be coregistered to the reference MR and the saved transformation matrices would subsequently be applied to bring all MR-CT pairs to the target’s coordinate system before applying voxel-wise weighting to estimate the pseudo-CT’s HUs [186]. This method reduces the computation time, which was mainly due to the multiple registrations. It outperformed the Dixon sequence and led to errors of up to 8% for all tissues in the reconstructed PET images when compared to reconstruction using a CTAC. In pelvis, Wallsten et al. [187] performed a similar method to the “template” approach described above [172, 173] but used machine learning to determine the weights applied on each voxel of CT images comprising the atlas. Alternatively, Hofmann et al. used pattern recognition to find the patches from the MR-CT pair database that better correlate with the investigated patch from the target image [188, 189]. Although this approach performed very well in most organs with errors of up to 8% in SUV values, the corresponding error in lung was up to 30% high with subsequent attempts to improve the method having moderate effects on the overall decrease on the SUV biases [190].

All atlas-based AC methods that have been applied on clinical PET data and report relative agreement with a reference method are listed in Table 3.

**Table 3:** List of original atlas methods evaluated on clinical PET data

Study	Method	Validation data† (tracer)	Region	Reported error (%)	
				Proposed method	Vendor method
Kops et al. [166]*	Generation of pseudo-transmission images by bringing the averaged database images to the patients’ space	4 (2-[^18^F]FDG)	Brain	9	N/A
Hofmann et al. [188]	Generation of pseudo-CTs using pattern recognition to find similar patches between a brain MR-CT pair database and target MRI	3 (N/A)	Brain	3.2 ± 2.5 (VOIs in brain)	N/A
Malone et al. [167]*	Generation of pseudo-transmission images by registering a brain MR-transmission maps database to the patients’ *T*_1_	10 (2-[^18^F]FDG)	Brain	−1.2 ± 1.2	N/A
Wollenweber et al. [170]	Pseudo-CT generation by registration of patient’s *T*_1_ image to a CT database	13 ([^18^F]FDG)	Brain	0.2 ± 2.6 (VOIs in brain)	N/A
Arabi et al. [190]	Adaptation of [188] using voxel-wise local normalised cross-correlation	14 (2-[^18^F]FDG)	Whole Body	~ 5 ± 4 (cerebellum), ~ 4 ± 10 (lung)	N/A
Burgos et al. [60]	Generation of pseudo-CTs by registering a MR-CT pair database to patients’ *T*_1_ image a using local image similarity measure	41 (2-[^18^F]FDG)	Brain	0.2 ± 2.1	UTE: −11.8 ± 2.1
Izquierdo-Garcia et al. [172]	Generation of 6-class probability maps from patients’ *T*_1_ image and coregistration to a similarly created probability map database with paired CT images. Averaged CT is brought back to patients’ space	15 (2-[^18^F]FDG or [^18^F]FET)	Brain	−1.0 ± 2.5	Dixon: 13.0 ± 10.2
Poynton et al. [173]	Registration of 3-class probability maps from CT data-dUTE-*T*_1_ database to patients’ corresponding MR images to generate a tissue probability map	13 (N.A.)	Brain	1.8 ± 2.4^	N/A
Chen et al. [181]	Generation of pseudo-CTs by registering a MR-CT pair database to patients’ *T*_1_ image. Air segmentation is performed using an air probabilistic map and sparse regression to identify similar patches between atlas and patients’ MR	20 ([^18^F]Florbetapir)	Brain	2.4 ± 1^	Dixon: 12.7 ± 2.2^
Burgos et al. [177]	Use of convolution-based local normalised correlation coefficient to irregular regions to account for mismatches in the FOV between CT and MRI and extension of the database to include both *T*_1_ and *T*_2_ images	15 (2-[^18^F]FDG or [^18^F]Florbetapir)	Brain	1.6 ± 0.6 (2-[^18^F]FDG), 1.9 ± 0.6 ([^18^F]Florbetapir) ^□^	N/A
Arabi et al. [186]	Pseudo-CT generation by registering an averaged atlas image to the patients’ *T*_1_ and use the precomputed transformation matrices to bring all atlas images to the patients’ space	41 (2-[^18^F]FDG)	Whole body	3.5 ± 6.1 (cerebellum), 3.2 ± 9.8 (lung), −4.9 ± 5.9 (bone)	N/A
Teuho et al. [174]	Assignment of μ values on a tissue probability map generated from *T*_1_ images in SPM	7 (2-[^18^F]FDG)	Brain	−0.3 ± 2.7 ^□^	UTE: −7 ± 2.3^□^
Wallstén et al. [187]	Statistical decomposition algorithm to generate pseudo-CTs from the patients’ *T*_2_ images	15 ([^11^C]acetate)	Pelvis	−2.3(prostate lesion) −4.2 ± 5.7 (bone)	Dixon: −5.6 Dixon: −17.7 ± 8.4
Sousa et al. [165]*	A database of paired of ^68^Ge-transmission AC maps-T1 images registered to the patients’ *T*_1_ image and averaging all transmission AC maps	9 ([^11^C]PE2I)	Brain	−0.1 ± 3.2	N/A

The regional mean relative error along with the standard deviation (where available) in radiotracer uptake across subjects is reported unless otherwise specified. The corresponding error for the vendor-provided method is quoted where available. CT was used for reconstruction of the reference images unless otherwise specified

^†^Number of patients on which the method is evaluated, *transmission data used for reconstructing the reference PET images, ^ relative absolute error is reported, ^□^ voxel-wise error is reported

### Discussion

As was the case with previous sections, the atlas-based methods literature is mainly focused in developing a method which outperforms the vendor-provided Dixon and UTE sequences. Nonetheless, a handful of studies provide a bit more insight into how the different techniques compare. As expected, the relatively “outdated” single-atlas method, which collapses to a simple coregistration problem without taking into account the intra-subject variability, was easily outperformed by the dual-atlas method approaches [179]. On the other hand, to take full advantage of the multi-atlas methods, a large diverse database is required to achieve an accurate registration between the atlas and the target’s images. This makes the method more applicable in the head as its size and shape is less variable when compared to the organs in the thorax for example. Even for the head though, it has been shown that an adult database might not be suitable for a paediatric cohort and vice versa [33, 191]. In addition, MacKewn et al. demonstrated that even in the case of patients with thick hair (which is not included in the atlas databases) up to 10% bias might be observed at the occipital part of the brain [192].

In terms of accuracy, most methods seem to provide less than 5% bias when compared to CT attenuation correction in the brain. Cabello et al. reported similar results when comparing the methods proposed by Burgos et al. [60] and from Izquierdo-Garcia et al. [172] with a slightly higher intersubject variability for the latter. Similar conclusion was reported by Ladefoged et al. [36], who compared the methods proposed by Burgos et al. [60], Izquierdo-Garcia et al. [172] and Merida et al. [178] with all three methods having similar performance in the brain and all of them outperforming the vendor-provided Dixon and UTE sequences and the MLAA method. More specifically, the methods from Burgos et al. [60] and Merida et al. [178] performed better in terms of bone accuracy while the methods from Izquierdo-Garcia et al. [172] and Merida et al. [178] had the lower variability in the cerebellum.

Only a limited number of studies have extended the atlas methods for whole-body applications [186, 188, 190]. Unfortunately, these studies indicate that these methods provide only moderate improvements when compared to a Dixon-based attenuation correction including bone information.

A generic disadvantage of all the atlas-based methods is the complexity in implementing them. Most methods require offline post-processing with the overall runtime for implementing them taking between 30 min and 2 h or more [36], making it impractical for a clinical setting [193]. The fact that most methods need offline post-processing also means that access to additional tools is required, making it a multi-step procedure. Pitfalls surrounding such procedures include standardisation of the offline tools used for coregistration, for the extraction of tissue probability maps and to make sure that the methods are streamlined and do not depend heavily on the user. Moreover, most methods require at least one anatomical image to be acquired for the atlas to be transferred to. This means that an acquisition of 5–6 min is required for each bed position. Even though in most research studies and in the brain, this is generally not an issue, in a clinical setting where patients scanned with 2-[^18^F]FDG for less than 4 min/bed position, this might be a limiting factor.

Considering the similarity in accuracy that most of these methods provide, it would make sense to opt for the most straightforward and easier to implement. The methods proposed by Burgos et al. [60], Merida et al. [178] and from Izquierdo-Garcia et al. [172] are all of similar complexity and seem to be leading to comparable results and are probably more easily adapted for body applications [194].

## Machine learning attenuation correction methods

Although the majority of publications for attenuation correction on PET-MR in the last three years are dominated by deep learning methods, a few earlier studies used “traditional” machine learning to generate pseudo-CT images. Those are more user dependant, as structured data need to be generated from the images and be used as input to train a clustering algorithm such as Gaussian mixture model, support vector machine or random forests. With additional input from the user when the outcome is sub-optimal, the algorithm can then quickly process new data. These methods do not require high computational power, but they need a large amount of data for accurate tissue classification. Deep learning is a subset of machine learning, which quickly became popular thanks to the recent technological advancements making powerful graphic processing units widely available, and the availability of large databases that can be implemented for training deep learning models. These will be simply referred to as “deep-learning methods” for the rest of this review to differentiate them from the machine learning methods. One of the main differences compared to machine learning is that deep-learning is less user-dependant as the algorithms rely on training their artificial neural networks to identify underlying features in the images while learning from their own errors. Therefore, these methods have no need for “hand-crafted” data.

### Machine learning methods

Machine learning methods have been used widely in the effort to perform attenuation correction. However, this review will only describe methods in which machine learning is the predominant method rather than peripherally being applied in the methods described above. One of the earliest approaches was presented by Johansson et al., who used two UTE image sets, a *T*_2_ image and a CT image from just four brain scans. A Gaussian mixture regression model was then used to link the intensities between MR and CT images in order to predict the pseudo-CT from an MR input [195, 196] with a number of studies also adapting this method [197, 198] or using polynomial regression [108] and support vector regression instead [199].

Most commonly though, manually extracted features from paired MR and CT images such as the spatial coordinates, pairwise voxel differences [200], gradient, textural and special frequency features [201–203] are used to identify regions of the same class. One of the few such approaches applied on brain 2-[^18^F]FDG PET data, incorporated random forest regression to generate the pseudo-CT leading to biases of up to 4% [201, 202].

Alternatively, a few groups employed machine learning methods to NAC PET data [204] or on the refinement of the existing MR-based AC methods [205] in order to avoid the need of additional datasets from another scanner although the methods are still to be applied on PET data.

### Deep learning methods

The generic principle in deep learning is to define a neural network and train the algorithm on paired data to predict the target image when given an input image or images. The training process broadly resembles the iterative reconstruction process with the data first being forward-propagated and applied to all neural layers until the final prediction is generated. A loss function is applied to evaluate the accuracy of that prediction, the loss is then back-propagated in order to fine-tune the weights and the process is repeated, undergoing an iterative procedure until the loss-function is minimised [206]. The three main steps for deploying an algorithm involve: (1) the training part using the input and target images while withholding a subset of the initial data from the database, (2) validation of the performance of the model while fine-tuning the hyper-parameters and (3) testing of the algorithm using an external dataset.

Despite the difficulty in finding a meaningful relationship between CT and MR images using traditional techniques, deep learning approaches, by identifying appropriate underlying features from both images, have been fairly successful in predicting CT from MR images. The majority of deep learning applications in this context make use of convolutional neural networks (CNNs). A popular sub-category of the CNNs, especially in the context of semantic segmentation, are the fully convolutional networks (FCNs), which mainly use convolutional operations between layers rather than including fully connected layers which result in reduced number of parameters and therefore faster training. Their general architecture is an encoding path in which the input image is encoded into features and a decoding path in which the features are used to predict the final image. The most popular algorithm currently is the U-Net, which was initially proposed for image segmentation in which information from the encoding part is passed onto the decoding part to regain lost spatial information [207]. A combination of the two previous methods would be the generative adversarial network (GAN) with their FCN model used as the generator and a CNN as the discriminator (adversarial) network which tries to discriminate between the true and pseudo-CT images as produced by the FCN model [208]. The encouraging results from such methods have resulted in a large number of studies trying to address the problem of attenuation correction in PET-MR for both brain and body acquisitions.

#### Brain

##### U-Net

As mentioned earlier, the majority of deep-learning-based AC for both brain and non-brain methods employ the U-Net architecture. The main differences between the methods adopting the U-Net algorithm are the architecture of the encoding path, the type of data used (2D or 3D) and the type of input and ground-truth (output) images. The vast majority of these studies aim at creating images with continuous values rather than performing classification tasks for attenuation correction.

Perhaps the most intuitive approach in terms of the data provided to the network is paired CT and anatomical MR data [209–211]. Paired UTE [210, 212, 213], Dixon [214, 215], ZTE-based [75] and *T*_1_-weighted [215, 216] with CT images have been used to train the algorithm leading to comparable results, which in all cases outperformed the vendor-provided MR-based AC methods with SUV biases of approximately 5% [75, 210, 211, 214] for 2-[^18^F]FDG and has been evaluated for various other tracers as well such as [^11^C]PiB, [^18^F]MK-6240 [217] and [^15^O]H2O PET [213]. The combination of both ZTE and Dixon images as input data has not shown a significant improvement compared to a single set of input data [214] although the idea of using multiple MR images has not been extensively investigated. In addition, it has been shown that noisy images such as dynamic PET data can also be provided as priors to the network to extract low-level image statistics which could help to fine-tune the final prediction [218, 219].

Another intriguing concept is to avoid the use of anatomical MR images and use pairs of images whose signal is more correlated. Such examples are the NAC PET (input) along with their corresponding CT images (ground truth) [41], NAC (input) with CT-based AC (ground truth) PET images [220–223], and the MLAA-generated activity distribution and *μ*-map (input) along with the corresponding CT (ground truth) [224, 225]. Those methods exhibited higher biases in SUVs when compared to other deep learning studies. It should be noted though, that so far, only the 2D version of the network has been applied to the data (a single slice rather than multiple slices is used as input to the model) making it unclear whether the higher bias is due to that or the lack of paired structural images during the training process. Other methods are more difficult to replicate in most clinical settings [226].

A more recent technique that attempts to further improve the pseudo-CT is to incorporate the U-Net into a GAN architecture (although alternative pairs such as MR and corrected PET images have also been proposed [227]). The additional discriminator model in these architectures which compares the pseudo-CT as generated from the U-Net with its original image helps in refining the final image. GANs are therefore recommended for complex tasks but are more difficult to train. However, using a 3D patch-based CNN structure as the discriminator in what is known as the cycleGAN (assess the generated pseudo-CT using the real CT and the generated pseudo-MR using the real MR), Gong et al. did not report notable differences compared to the U-Net when training 3D data [228].

##### Other networks

Contrary to the previous methods, many of the initial attempts aimed at identifying the various classes within the organ (soft tissue, bone, air, etc.) and applying a uniform μ value across that class. The most widely used network in this context is the VGG16, which uses 16 layers that contain weights in which each voxel of the input image is classified to predefined tissues classes. Coregistered paired CT images thresholded to three tissue classes and anatomical MR [114, 229, 230] or UTE [231] images have been used as training data for variations of the network. The corresponding MR image of the target could then be used as input to generate a pseudo-CT with uniform HUs for each predefined class. Although this approach hasn’t been extensively applied, a significantly reduced bias in SUV is reported for brain 2-[^18^F]FDG-PET scans compared to the Dixon method with biases of approximately 1% [114]. The longer training requirements of the network along with the fact that it results in uniform HUs for a certain number of classes, make the method less appealing. An alternative to VGG16, with comparable performance in the overall brain, is to use a three-layer probabilistic neural network which estimates the probability of the UTE images to belong in one of the specified classes [232, 233].

Several other networks have also been applied for generating pseudo-CT images with continuous values but have drawn limited attention so far. Of note is the GAN-based approach by Arabi et al. who used a structure of three convolutional and three fully connected layers for each set of GANs with the first set synthesising the pseudo-CT image (synGAN) and the second taking the pseudo-CT image and segmenting it into soft tissue, bone, air in cavities and air in background (segGAN) [38]. Another notable example is the high-resolution network (HighRes), which was first introduced for image segmentation [234]. The network starts from high-resolution convolution streams (blocks) adding high-to-low convolution streams while moving deeper in the network. The various blocks are connected in parallel to maintain the information of the high-resolution information. Variations of this network have been trained to either generate pseudo-CTs from anatomical *T*_1_ and *T*_2_ images [235, 236] or to generate *μ*-maps from sinogram data [237] with both attempts leading to fairly accurate PET images. Other promising approaches, which have resulted in images comparable to ground-truth CT images, have yet to be applied to PET data for attenuation correction [208, 238–240].

#### Whole-body

##### U-Net

As in previous sections, the studies applying deep learning methods in body images are more limited compared to brain. Moreover, since most deep learning studies applied in body regions are relatively recent, the majority are attempting to predict the value of the output image at the pixel level. Deep learning methods using anatomical paired MR-CT images as input have mainly been used in the pelvis which is less prone to motion compared to thorax. The challenge in this case is to accurately identify the bone which is where most MR-based techniques are prone to error. A number of methods which used paired 2D Dixon and CT [241, 242], 3D ZTE and CT [40], 3D *T*_1_ and CT [243] images or an additional deep learning-based segmentation step to segment the air from the bowl areas [244], resulted in comparable biases of approximately 5% in the pelvic bone regions. Moreover, it was recently shown that if the uncertainty in the prediction is also taken into account, implants could be more easily identified [245, 246].

In studies that involve regions prone to involuntary motion, most techniques tried to avoid the use of paired MR and CT images, mainly to circumvent the need of data from another modality. When anatomical images were used, non-rigid registration between the input data was performed before providing them to the network. In order to bypass the registration problem, Dong et al. used NAC 2-[^18^F]FDG PET images to predict the attenuation-corrected image in the cycleGAN network [228, 247–251]. In another noteworthy study from Guo et al., the low-frequency information was used from the AC and the NAC PET images, which were more indicative of the anatomy rather than the tracer distribution, from which the correction map was estimated and used to make predictions more generalisable for different tracers [252]. In other methods, coregistration had to be performed between the MLAA-generated activity distribution and *μ*-map with the corresponding CT [253–257], the NAC PET and the CT images [258–264], and in a more recent study, the reconstructed PET image was predicted directly from paired *T*_1_ and PET images as reconstructed with the vendor-provided method [265]. In all those methods, the results for the lung are much improved compared to the Dixon method even for low-dose data [257]. However, the reported errors are still approximately 10%, indicating that further improvements could still be performed. Moreover, the main drawback is that these methods are specific to the tracer in the PET images used for training. Nonetheless, despite difficulties in coregistrations, Schaefferkoetter et al. reported similar levels of bias when using the cycleGAN to predict pseudo-CT from Dixon images [266].

##### Other networks

One of the few attempts to generate a pseudo-CT with a classification method was also one of the earliest by Nie et al. who fed paired *T*_1_ and CT images of the pelvis to a relatively shallow 3D FCN achieving a good agreement with the ground-truth CT although a PET evaluation was not performed [267, 268]. The most notable example though is the one from Bradshaw et al. [269], who used the DeepMedic architecture [270]. The network consists of two blocks of convolutional layers each ran in parallel, with one block receiving patches of normal and the other of low resolution *T*_1_ and *T*_2_ images from the pelvis, followed by two fully connected and a classification layer. As in previous studies, in order to avoid registration of the input data, a synthetic CT image with uniform HUs for each class and generated by combining the Dixon, *T*_2_ and CT images was used as ground truth. When applied in the pelvis though, similar or higher level of bias was reported when compared to previously described deep learning methods. Moreover, the HighRes method has also been applied in the torso with extremely promising results [271, 272].

All deep learning-based AC methods that have been applied on clinical PET data and report relative agreement with a reference method are listed in Table 4.

**Table 4:** List of original deep learning methods evaluated on clinical PET data

Study	Method	Training / validation / testing data† (Tracer)	Region	Reported error (%)	
				Proposed method	Vendor method
Ribeiro et al. [233]	Generation of template-based *μ*-maps [168] from UTE images using a three-layer network	4/N.A/N.A (2-[^18^F]FDG)	Brain	3.4	N/A
Bradshaw et al. [269]	Generation of 4-class probability maps from *T*_1_ and LAVA Flex images using the 3D DeepMedic network	12/6/N.A (2-[^18^F]FDG)	Pelvis	− 1.0 ± 1.3 (lesions)	Dixon: 0.0 ± 6.4
Gong et al. [214]	Generation of pseudo-CTs from Dixon and ZTE images using a 2D U-Net with group convolutional modules	32/8/N.A (2-[^18^F]FDG)	Brain	~ 2.0 ± 0.5 ^	*Dixon_SB_: ~ 5.5 ± 1.5 ZTE: ~ 4 ± 1.3
Jang et al. [231]	Generation of 3-class from rapidly acquired UTE images using a 2D VGG16 (encoder) and SegNet (decoder)	30 T1 + 6 UTE (transfer learning)/ N.A/ 8 (2-[^18^F]FDG)	Brain	0.2 ± 1	N/A
Liu et al. [229]	Generation of 3-class pseudo-CTs from AC PET 2-[^18^F]FDG images using a 2D VGG16	100/28/N.A (2-[^18^F]FDG)	Brain	−0.6 ± 2.0 ^□^	N/A
Liu et al. [114]	Same as [229] but using a 13-layer VGG16 with *T*_1_ images as input	100/28/N.A (2-[^18^F]FDG)	Brain	−0.7 ± 1.1 ^□^	Dixon: −5.8 ± 3.1
Arabi et al. [38]	Generation of 4-class *μ*-maps from *T*_1_ images using the 3D adversarial semantic structure	15/15/N.A (2-[^18^F]FDG)	Brain	3.2 ± 3.4 (whole head) ^□^	N/A
Blanc-Durand et al. [75]	Generation of pseudo-CTs from ZTE images using a 3D U-Net algorithm	50/43/N.A (2-[^18^F]FDG)	Brain	−0.2 ± 5.6 ^□^	ZTE:2.5
Dong et al. [247]	Generation of pseudo-CTs from NAC PET images using a 3D cycleGAN	80/39/N.A (2-[^18^F]FDG)	Whole body	− 1.1 ± 3.9 (Brain), 10.7 ± 7.7 (Lung), 0.7 ± 8.4 (Heart)	N/A
Hwang et al. [253]	Generation of CT-based *μ*-maps from MLAA images using a 2D U-Net	60/20/20 (2-[^18^F]FDG)	Whole body	− 2.2 ± 1.78 (bone lesions) 1.3 ± 3.3 (soft-tissue lesions)	Dixon: −9.4 ± 5.2 Dixon: −2.9 ± 1.2
Ladefoged et al. [212]	Generation of pseudo-CTs for paediatric data from UTE, echo images and the *R*_2_^*^ map using a 3D U-Net	60/19/28 ([^18^F]FET)	Brain	−0.1	N/A
Shiri et al. [220]	Generation of AC from NAC PET 2-[^18^F]FDG images using a 2D U-Net algorithm	91/20/18	Brain	− 0.1 ± 2.14	N/A
Spuhler et al. [226]**	Generation of pseudo-transmission data from a spoiled gradient recalled acquisition using a 2D U-Net	66/11/11 ([^11^C]WAY-100635) + 10 ([^11^C]DASB)	Brain	− 0.5 ± 1.7 ([^11^C]WAY-100635) − 1.5 ± 0.7 ([^11^C]DASB) ^□^	N/A
Torrado-Carvajal et al. [241]	Generation of pseudo-CTs from Dixon images as input to a 2D U-Net algorithm	15/4/N.A (2-[^18^F]FDG)	Pelvis	0.3 ± 2.6 (Fat), − 0.0 ± 3.0 (soft tissue), − 0.9 ± 5.1 (bone)	Dixon: 1.5 ± 6.5 Dixon: −0.3 ± 10.0 Dixon: −25.1 ± 12.7
Arabi et al. [237]	Generation of CT-derived pseudo-*μ*-maps from PET TOF sinogram data using the 2D HighRes framework	52/16/N.A (2-[^18^F]FDG)	Brain	2.9 ± 3.1 (head), 2.0 ± 10.6 (soft tissue), 1.2 ± 10.2 (bone)	N/A
Armanious et al. [259]	Generation of pseudo-CT from NAC PET 2-[^18^F]FDG images using a 2D GAN framework	100/N.A/25 (2-[^18^F]FDG)	Whole body	− 5.6 ± 7.2 (left lung), − 3 ± 11.7 (right lung)	N/A
Dong et al. [248]	Generation of AC from NAC PET images using a 2D cycleGAN framework	25/1 (leave-one-out)/30 (2-[^18^F]FDG)	Whole body	− 17.0 ± 12.0 (lung), 2.1 ± 2.5 (heart), 2.8 ± 5.2 (lesions) ^□^	N/A
Hu et al. [258]	Generation of pseudo-CT and AC from NAC PET images using a 2D Wasserstein GAN	40/5/N.A (2-[^18^F]FDG)	Whole Body	6.4 ± 3.8 (brain), 4.4 ± 3.2 (heart), 4.3 ± 5.4 (lung) ^□^	N/A
Ladefoged et al. [215]	Similar to [212] but using DIXON images as input	403 + 5 (transfer learning)/207/104 (2-[^18^F]FDG)	Brain	~ −0.3	Dixon_SB_: 0.8 ± 2.4
Anaya et al. [303]***	Generation of pseudo-CT from Dixon water images using the (2D) pix2pix framework	9/2/1 (N/A)	Head & neck	2.1^^^	N/A
Chen et al. [210]	Generation of pseudo-CTs from R1 images as derived from UTE using a residual 3D U-Net	72/18/84 ([^18^F]Florbetapir)	Brain	0.1 ± 0.6 ^□^	N/A
Choi et al. [225]	Generation of tracer-specific pseudo-*μ*-map from MLAA images using a 3D U-Net	60/20/20 (2-[^18^F]FDG)	Brain	< 5	N/A
Gong et al. [217]	Same as [214] but using images from a UTE/multi-echo sequence as input	30/5/N.A ([^11^C]PiB, [^18^F]MK6240)	Brain	< 2	N/A
Gong et al. [228]	Generation of the pseudo-CTs from Dixon images using a 3D cycleGAN	28/4/N.A ([^18^F]FDG)	Brain	~ 3	Dixon: ~ 8
Hashimoto et al. [41]*	Generation of pseudo-CTs from NAC PET images using a 2D U-Net algorithm and mixed tracer training data	1091/N.A/70 (6 tracers)	Brain	− 5.7 ± 5.0 (2-[^18^F]FDG)	N/A
Kläser et al. [236]	Pseudo-CT generation from *T*_1_ & *T*_2_ images using HighRes3DNET and imitation learning	16/4/23 (2-[^18^F]FDG)	Brain	4.04 ± 0.5 ^^□^	N/A
Pozaruk et al. [243]	Generation of pseudo-CTs from Dixon images using a 2D cycleGAN framework	18 /N.A/10 ([^68^ Ga]Ga-PSMA)	Pelvis	2.2 ^^□^	Dixon: 10.3, Dixon_SB_: 8.7
Shiri et al. [305]	Generation of tracer and sight-specific AC from NAC PET images using a 2D U-Net and transfer learning techniques	1110 (2-[^18^F]FDG) & 855 ([^68^ Ga]Ga-PSMA)/N.A./95 ([^68^ Ga]Ga-PSMA)	Whole body	2.72 ± 7.5	N/A
Ahangari et al. [242]	Generation of pseudo-CT from Dixon images using a 3D U-Net	11 (transfer learning)/N.A./15	Whole body	2.1 ± 2.4 (Brain), − 4.9 ± 12.1 (lung), − 4.0 ± 6.5 (bone)	Dixon_SB_: 2.1 ± 3.2, Dixon_SB_: −4.3 ± 20.3, Dixon_SB_: −7 ± 12.4
Arabi et al. [271]	Pseudo-CT generation from in-phase Dixon images using the 2D HighResNet	20/5/N.A (2-[^18^F]FDG)	Whole body	− 3.7 ± 5.5 (lung), 1.1 ± 3.1 (bone), 2.1 ± 3.7 (cerebellum)	N/A
Hwang et al. [306]	Generation of tracer-specific pseudo-*μ*-map from MLAA images using a 3D U-Net	60/20/20 (2-[^18^F]FDG)	Whole body	1.2 ± 5.7 (lung lesions), 0.2 ± 3.8 (bone lesions)	N/A
Olin et al. [211]	Generation of pseudo-CTs from Dixon images using a 3D U-Net	800 heads + 17 head & neck (transfer learning)/leave-one-out/10 (2-[^18^F]FDG)	Head & neck	− 0.6 ± 2.0 (lesions)	Dixon_SB_: −3.5 ± 4.6
Sari et al. [289]	Air pocket segmentation from Dixon images using a 3D U-Net followed by generation of pseudo-CTs from Dixon images using a second 3D U-Net	30/5/N.A (2-[^18^F]FDG)	Pelvis	2.6	Dixon_SB_: 5.1
Toyonaga et al. [255]	Generation of tracer-specific pseudo-*μ*-map from MLAA images using a 3D U-Net	40/22/73 (2-[^18^F]FDG) 40/22/36 ([^68^ Ga]DOTATATE) 40/22/50 ([^18^F]Fluciclovine)	Whole body	Thorax: − 1.5 ± 2.3 (2-[^18^F]FDG), 2.2 ± 2.3 ([^18^F]Dotatate), − 2.9 ± 1.8 ([^18^F]Fluciclovine)	N/A
Wang et al. [307]	Generation of AC from NAC PET images using a 2D U-Net with deformable transformer layers	21/4/5 (^13^N-Ammonia)	Thorax	10.1 ± 2.9 (myocardium)	N/A
Shiri et al. [275]	Training of a “global” model for multi-centre trials, by feeding sight-specific trained models to it. AC from NAC PET images using a 2D U-Net are generated	180/60/60 (2-[^18^F]FDG)	Whole body	-0.1 ± 0.1	N/A

The mean relative error along with the standard deviation (where available) in radiotracer uptake for the whole region is reported unless otherwise specified. CT was used for reconstructing the reference images unless otherwise specified

^†^Number of patients used for training, validating and testing the model

*Dixon Segbone method (Dixon_SB_) [31]

**Transmission data used for reconstructing the reference PET images

***An atlas method [71] used for reconstructing the reference PET images, ^ relative absolute error is reported, ^□^ voxel-wise error is reported

### Discussion

The majority of studies that use “traditional” machine learning methods lack quantitative evaluation on reconstructed PET images and the limited available results, even though they indicate a relatively good agreement with the gold standard methods, do not lead to much lower bias when compared to the more established atlas methods. In addition, they could be equally time-consuming to implement making it challenging for a busy clinical environment. Deep learning techniques, on the other hand, are more appealing as they seem to provide accurate results while being quick to implement once the model is trained and deployed. Although the U-Net architecture is the one most widely used, the reported bias is of similar level for all studies. To properly compare the different methods, especially considering the limited number of quantitative PET evaluations for each method, the same training, validation and testing data would need to be used.

As in previous sections, the vast majority of the published studies are focused on the brain. The agreement with CT reconstruction seems to be quite impressive with most studies reporting a bias of up to 5%. Higher biases are quoted for studies which trained the network on 2D datasets. This highlights the need to utilise as much spatial context as possible in all dimensions [225]. The main advantages of the deep learning methods in terms of accuracy seem to be noted in the non-brain studies. Even though more limited in numbers, the reported bias in organs hampered by involuntary movement is considerably less when compared to MR, emission and atlas methods. The most intriguing approach for whole body studies would be the methods where no registration is needed for the input data to avoid misregistration errors as briefly mentioned in the motion correction section. However, if CTAC is used as a gold-standard which requires registration to the PET data, it might be difficult to evaluate their accuracy [259]. Moreover, since the networks learn to some extent the biodistribution of the tracer used in the non-corrected/corrected images, they might not be generalisable to any tracer.

One of the limiting factors in the majority of published studies is the lack of testing on external datasets with the validation data being used instead for assessing the performance of the method [273]. This is a general issue in the field of AI and deep learning that could lead to “data leakage”. Kapoor and Narayanan recently evaluated the reproducibility of various machine learning methods across different fields and reported issues to a staggering number of 329 studies whose results could not be replicated [274]. This strongly highlights the need for a rigorous assessment and standardised procedures when developing an algorithm. As standardisation strategy in multi-centre trials, Shiri et al. suggest the use of a single model that has been refined from the respective model trained for a single sight [275]. Moreover, similarly to the atlas methods, limited and non-diverse training datasets will have a direct effect on the generated output. Ladefoged et al. had to train paediatric only brain images as an adult database could lead to large errors [212]. However, their most recent work indicated that when applying transfer learning even with very small number of data the robustness of the model can increase and be applicable for brains of various sizes, different pathologies and even when metallic implants are present [215, 276]. Alternatively, simulated images could potentially improve the robustness of the network [277]. Finally, even though most studies in Table 4 report very small errors, a couple of recent studies have reported that a minimum of 100 training datasets are needed to generate a robust model that produces accurate pseudo-CT images [215, 278]. The amount of data usually available and restrictions in data sharing make such tasks challenging for most research centres. It is expected that this difficulty may be overcome with the increase of available public databases.

## Alternative attenuation correction methods

A handful of methods that fall outside of the aforementioned categories have also been proposed. A straightforward idea would be to simply use the NAC PET data and apply intensity thresholds in order to identify the various tissue classes from which the final *μ*-map could be generated [279]. Despite this method being appealing due to its simplicity and being independent of additional scans, certain structures such as the bone are still difficult to identify on an 2-[^18^F]FDG scan and it assumes certain biodistribution of the tracer. Another method, would be to use a [^18^F]NaF PET scan to identify the bone region which can then be segmented and added to the *μ*-map. Although this method does provide an accurate bone region, it still has the limitation that the patients need to undergo an additional [^18^F]NaF scan [280]. The idea of a transmission source has also been suggested with or without the combination of the existing attenuation correction techniques on PET-MRI [281–286] with promising results. All these methods require additional hardware to accommodate the transmission source which adds a level of complexity in the scanning process [286], and therefore, application to clinical data has been somewhat limited. An interesting approach by Rothfuss et al. is the use of the naturally occurring background radiation from the Lutetium Oxyorthosilicate (LSO) crystals for transmission scanning [287, 288]. The method has even been coupled with deep learning approaches to further refine the transmission image [289]. This still involved a few practical issues though as the patients needed to have the transmission scan prior to injection so that no additional radiation interferes with the scan.

## Attenuation correction of MR coils

Whilst the attenuation due to MR coils in the PET field of view occupies a much smaller percentage of the literature compared to human attenuation correction, it remains an important and active topic of research. Eldib et al. have previously presented a comprehensive review of the challenges and general methods for coil attenuation correction [290]. In brief, ignoring the MR coils during attenuation correction could result in an activity underestimation of up to 25% and visible artefacts on the reconstructed PET images [100, 291–294]. This problem is easier to tackle for rigid coils such as for the head and neck, as these remain in a fixed position during the scan. Therefore, one of the methods described in this review can be used to generate the human attenuation correction map, while a “template” of the attenuation map of the coil can retrospectively be added to it before the final composite map is used for reconstruction of the PET data [103, 290]. This “template” can be a CT scan [100, 103], a transmission scan [283, 284], a computer-aided design of the coil [105] or transmission data using background radiation from the LSO crystals [295], with all methods being able to reduce the activity bias to less than 5%. Using CT scans is the most straightforward and easily accessible approach and has been used to include other rigid hardware as well such as radiotherapy flat-beds [296], while it is also the method currently implemented by the manufacturers. Issues such as streaking artefacts due to metallic components have been easily addressed by simple thresholding, while the bilinear interpolation method to convert HUs to linear attenuation coefficient at 511 keV has been found to be applicable even for those highly attenuating components [3, 296]. Nonetheless, the level of accuracy could vary by a factor of two depending on the coil used [297] while coils with many metal components could still lead to substantial artefacts [290]. Moreover, accurate registration approaches need to be followed since even a 2 mm misregistration in the interface between the head and neck coil could lead to visible artefacts [100].

This problem becomes much more challenging for the flexible coils used for body scans as these are not in a fixed position and adapt to the patient’s body shape. These coils are currently not taken into account when performing attenuation correction on PET-MR scanners. Most approaches rely on performing a CT scan of the coil and then try to localise it on the MR images in order to coregister the CT to the MR image [290]. The localisation of the coil can be performed by using fiducial markers [291, 292], a UTE sequence [103, 290] and more recently, with a camera that is able to provide 3D information of the imaged object (Kinect V2) [294]. A workaround for radiotherapy studies on the pelvis, is to set-up a rigid coil-holder to place the coils on top and then follow a similar approach as for the rigid coils [298]. Despite all methods showing decrease in bias, they also exhibit certain implementation difficulties [290, 294]. An interesting approach suggested by Heuẞer et al., which still needs to be evaluated against a reference method, is the use of the MLAA algorithm with the attenuation being updated only outside of the patients’ with a fixed AC map being given for the patients’ body [293].

Ideally, a holistic approach that addresses the attenuation from all materials in the FOV of the PET-MR scanner would be used. However, the attenuation of coils, due to their inability to produce a MR signal, is studied independently to the human attenuation correction. Since the main source of attenuation has been shown to be mainly due to the casing of the coils though [100], perhaps the future direction for at least the mitigation of this problem might be the design of new low attenuating coils with a few studies already suggesting designs that could reduce PET quantification bias to less than 5% [299–301].

## Overall discussion

Despite the considerable number of developed methods for performing attenuation correction on the PET-MR, the problems has, unfortunately, not been fully addressed, and this is reflected by the large amount of ongoing research and number of new studies currently being published. One of the main reasons is the large level of bias in certain regions when the vendor available techniques are applied, which make it relatively easy to develop a method that outperforms them. Why do recent studies still tend to compare their methods with the Dixon- or UTE-based *μ*-maps even though it has been established that in most cases they are not as accurate or reproducible? We believe that the answer is twofold: (i) Despite their poor performance in terms of accuracy these methods remain the most straightforward and easy to implement with minimal user input which makes them attractive in a clinical setting and (ii) the overwhelming literature, which also tends to be region specific, has not allowed many methods to be widely established in order to be used as comparators when a new method is proposed while the vendor methods are readily available. Recent guidelines from the European Association of Nuclear Medicine (EANM) for clinical 2-[^18^F]FDG brain scanning also do propose the use of the vendor-provided MR sequences for attenuation correction until more advanced techniques such as deep learning are commercially available [302].

For PET-MR scanners to be finally introduced into clinic, an attenuation correction method with the following criteria are required:

- To be accurate and reproducible
- To provide images comparable to state-of-the-art PET-CT scanners
- To be quick and easy to implement without the need of specially trained staff

The following desirable criteria would also provide ease of use in PET-MR scanning

- To be generalisable (i.e. independent of tracer, patient age, etc.)
- To be independent of the scanned region
- To be insensitive to registration errors between PET and attenuation correction map

The advantages, disadvantages and a summary of the characteristics of the four approaches discussed in this review are summarised in Table 5.

**Table 5:** Comparison of the four attenuation correction techniques outlined in this review

	MR-based AC	Emission-based AC	Atlas-based AC	Deep learning-based AC
Accuracy	Low but somewhat improved when properly incorporating bone tissue [36, 73]. Low accuracy in lung and pelvis [120, 241, 308]	Good accuracy in the brain and tissue lesions. Moderate accuracy in air cavities, bone and lung [36, 142]	Good accuracy in the brain for most methods [36]. Moderate in whole-body (limited number of studies) [6]	Good accuracy in brain and body [242, 271]
Artefacts and biases	Truncation artefacts [26]. Motion artefacts in lung and heart [22] Metallic artefacts when implants are present [47]. Workaround techniques to partially alleviate the artefacts [49, 97]	Additive constant [132]. Positive bias on low count data [133]. Crosstalk in non-TOF data [131]	Moderately sensitive to metallic and truncation artefacts [271]. High biases for non-standard anatomies [10, 192]. Separate adult and paediatric databases required [33]	Insensitive to metallic and truncation artefacts [271]. Separate adult and paediatric network training may be required [212]. Insensitive to tracer (depending on the technique) [228, 255]
Processing time	~ 20 s—10 min depending on the sequence [52, 109]	~ 1 h [253]	30 min—several hours [167, 185]	Few seconds—few minutes [242, 271]
Provided by the manufacturers	Yes	No. Only for addressing truncation artefacts on the Siemens mMR [158]	One method that requires *T*_1_ images is available on the GE SIGNA [170]	No
User input	Minimal. Only the acquisition of the sequences required	Moderate/High. Increases when coregistration is also required	Moderate/High. Data acquisition, potential data processing and visual inspection	Moderate. After training only the trained weights need to be applied on the new image
Susceptibility to misregistration	Yes [14]	Not for the original MLAA. Yes for methods requiring anatomical priors or initial μ values [137]	Yes. Most methods require two or more registration steps [43, 60]	Yes, if an anatomical MR image is used as input [247]
PET dependency	Independent	Not suitable for non-TOF systems and low count datasets [130]	Independent	Independent if anatomical images are used. Could be tracer-dependent if NAC images are used as input [255]
Applicability to whole-body	Only Dixon-based sequences [67]	Yes [142]	Separate atlases for each region. Not widely used in whole body [186]	Yes [242, 248]
Requirement for additional data	No	Tissue priors required to tackle the additive constant issue	MR images if they are used as input	MR images if they are used as input
Additional requirements	None	Coregistration and/or segmentation tools	Large database of paired CT and MR images Coregistration and/or segmentation tools	Large image database for model training on a powerful workstation or a suitable pre-trained model if available

If a region-specific approach is to be adapted, then there is probably not much value in investing more time in developing additional methods just for the brain. Many of the current methods, including the ZTE with continuous *μ*-values [73], a number of atlas methods [60, 172, 179, 181, 182, 193] and a number of deep learning methods [38, 210, 212, 228, 237, 239, 248, 303] have already demonstrated accuracy of less than 5% in most brain regions. Those would need to be compared in terms of the above criteria, and standardisation procedures need to be established if more than one is widely used.

Deep learning techniques seem to have been more widely applied in whole-body research patients compared to atlas- and emission-based techniques. The promising results in terms of accuracy, image quality and ease of use are the main contributors. Even research groups who had previously proposed atlas- and emission-based methods seem to be moving towards deep learning approaches. However, a rigorous evaluation of these methods is still required in terms of the above criteria. Many methods have not been tested against external datasets, which is an important evaluation step prior to model deployment as the model needs to be generalisable, i.e. to provide equally accurate results on independent patient cohorts. If further refinement is required, then it needs to be retrained using a more diverse dataset or use transfer learning methods.

In summary, thanks to the incredibly active research community which has deeply appreciated the importance of an accurate and robust attenuation correction method in PET, it seems that confidence in using PET-MR for clinical and research scanning is increasing, opening up the doors to the numerous applications that this modality can offer. However, a careful evaluation still needs to be performed for many of the proposed methods and the most accurate, robust and suitable for a clinical setting identified and if needed optimised.

## Data Availability

The datasets used and analysed during the current study are available from the corresponding author on reasonable request.

## Code availability

The code used for the generation of the figures in this study is available from the corresponding author on reasonable request.
